# Description of a Natural Infection with Decapod Iridescent Virus 1 in Farmed Giant Freshwater Prawn, *Macrobrachium rosenbergii*

**DOI:** 10.3390/v11040354

**Published:** 2019-04-17

**Authors:** Liang Qiu, Xing Chen, Ruo-Heng Zhao, Chen Li, Wen Gao, Qing-Li Zhang, Jie Huang

**Affiliations:** 1 Yellow Sea Fisheries Research Institute, Chinese Academy of Fishery Sciences; Laboratory for Marine Fisheries Science and Food Production Processes, National Laboratory for Marine Science and Technology (Qingdao); Key Laboratory of Maricultural Organism Disease Control, Ministry of Agriculture and Rural Affairs; Qingdao Key Laboratory of Mariculture Epidemiology and Biosecurity, Qingdao 266071, China; qiuliang@ysfri.ac.cn (L.Q.); chenxing910520@163.com (X.C.); ruohengzhao@126.com (R.-H.Z.); lichen@ysfri.ac.cn (C.L.); gaowen1994@163.com (W.G.); zhangql@ysfri.ac.cn (Q.-L.Z.); 2 Shanghai Ocean University, Shanghai 201306, China; 3 Dalian Ocean University, Dalian 116023, China

**Keywords:** DIV1, SHIV, CQIV, *Macrobrachium rosenbergii*, *Macrobrachium nipponense*, *Procambarus clarkii*, white head, susceptible species, viral load

## Abstract

*Macrobrachium rosenbergii* is a valuable freshwater prawn in Asian aquaculture. In recent years, a new symptom that was generally called “white head” has caused high mortality in *M. rosenbergii* farms in China. Samples of *M. rosenbergii*, *M. nipponense*, *Procambarus clarkii*, *M. superbum*, *Penaeus vannamei*, and Cladocera from a farm suffering from white head in Jiangsu Province were collected and analyzed in this study. Pathogen detection showed that all samples were positive for Decapod iridescent virus 1 (DIV1). Histopathological examination revealed dark eosinophilic inclusions and pyknosis in hematopoietic tissue, hepatopancreas, and gills of *M. rosenbergii* and *M. nipponense*. Blue signals of in situ digoxigenin-labeled loop-mediated isothermal amplification appeared in hematopoietic tissue, hemocytes, hepatopancreatic sinus, and antennal gland. Transmission electron microscopy of ultrathin sections showed a large number of DIV1 particles with a mean diameter about 157.9 nm. The virogenic stromata and budding virions were observed in hematopoietic cells. Quantitative detection with TaqMan probe based real-time PCR of different tissues in naturally infected *M. rosenbergii* showed that hematopoietic tissue contained the highest DIV1 load with a relative abundance of 25.4 ± 16.9%. Hepatopancreas and muscle contained the lowest DIV1 loads with relative abundances of 2.44 ± 1.24% and 2.44 ± 2.16%, respectively. The above results verified that DIV1 is the pathogen causing white head in *M. rosenbergii*. *M. nipponense* and *Pr. clarkii* are also species susceptible to DIV1.

## 1. Introduction

Globally, viral diseases have been acknowledged as a huge threat to the shrimp aquaculture industry. Among the viruses reported for crustaceans, *Cherax quadricarinatus* iridovirus (CQIV) and Shrimp hemocyte iridescent virus (SHIV) are two newly found viruses isolated from red claw crayfish *C. quadricarinatus* [1] and white leg shrimp *Penaeus vannamei* [2], respectively. CQIV and SHIV both have a typical icosahedral structure with a mean diameter of about 150 nm [1,2]. Evidence from histopathological study, transmission electron microscope (TEM) of ultrathin sections, and in situ hybridization (ISH) indicated that SHIV may mainly infect the hematopoietic tissue and hemocytes in gills, hepatopancreas, pereiopods, and muscle of *P. vannamei* [2]. Similarly, the TEM also showed that CQIV could infect hematopoietic tissue and gills in *C. quadricarinatus* and *P. vannameii* [1]. Phylogenetic analysis supported that SHIV and CQIV belong to a new genus, which was originally proposed to be named *Xiairidovirus* or *Cheraxvirus*, in the family *Iridoviridae* [2,3,4]. Alignment of the complete genomic sequences revealed that SHIV and CQIV might be different strains or genotypes of the same viral species [5]. In March 2019, the Executive Committee of the International Committee on Taxonomy of Viruses (ICTV) approved the proposal made by Chinchar et al. [6] that a new species of Decapod iridescent virus 1 (DIV1) in a new genus *Decapodiridovirus* to include SHIV 20141215 and CQIV CN01 as two isolates. We follow the ICTV’s decision to use the formally recognized name for general indication of the virus or newly identified strains, and SHIV 20141215 and CQIV CN01 for individualization of the original isolations. To date, DIV1 has been detected in farmed *P. vannamei*, *P. chinensis*, *P. japonicus*, *C. quadricarinatus*, *Procambarus clarkii*, *Macrobrachium nipponense*, and *M. rosenbergii* in China since 2014 [1,4,7], indicating that DIV1 is a new threat to the shrimp farming industry.

The giant freshwater prawn, *M. rosenbergii*, a valuable crustacean species in Asian aquaculture, are widely cultured in tropical and subtropical areas. *M. rosenbergii* is native to Malaysia and other Asian countries, including Vietnam, Cambodia, Thailand, Myanmar, Bangladesh, India, Sri Lanka, and the Philippines [8,9]. Being popular for its delicious flesh and high nutritional value, the global production of this species has increased from about 3,000 tons in 1980 to more than 220,000 tons in 2014 [10,11]. Generally, *M. rosenbergii* is considered less prone to some viral diseases in aquaculture when compared to penaeid shrimps [12]. Some viral pathogens, such as *Macrobrachium* hepatopancreatic parvo-like virus (MHPV), *Macrobrachium* muscle virus (MMV), Infectious hypodermal and hematopoietic necrosis virus (IHHNV), White spot syndrome virus (WSSV), *Macrobrachium rosenbergii* nodavirus (MrNV), and Extra small virus like particle (XSV), have been reported in prawns [13]. Recently, results of RT-LAMP and histopathological examination indicated that *M. rosenbergii* could be infected with Covert mortality nodavirus (CMNV) [14]. To date, only PCR results showed that cultured *M. rosenbergii* were DIV1 positive [2,7] and more pathological information is not available.

In recent years, a new symptom that occurred in *M. rosenbergii* farms in China has been commonly called “white head” or “white spot”, due to the diseased prawn exhibited a typical white triangle under the carapace at the base of rostrum [15]. Moribund prawns resting on the bottom in deep water and dead prawns can be found every day, with a cumulative mortality up to 80%. It is noteworthy that many *M. rosenbergii* populations suffering from white head were polycultured with *P. vannamei* [16]. In the present study, we investigated a diseased polyculture pond with *M. rosenbergii* and *Pr. clarkii*. In the pond, most of the *M. rosenbergii* exhibited typical the white triangle under the carapace at the base of rostrum and appeared moribund or died. One month before we arrived, all of the *P. vannamei* in an adjacent pond had died. Samples were collected and analyzed in this study.

## 2. Materials and Methods

All the protocols of animal handling and sampling were approved by the Animal Care and Ethics Committee, Yellow Sea Fisheries Research Institute, Chinese Academy of Fishery Sciences, and all efforts were made to minimize the suffering of animals according to recommendations proposed by the European Commission (1997). The study was carried out in accordance with the approved protocol. All the methods were applied in accordance with relevant guidelines.

### 2.1. Samples

Samples of farmed *M. rosenbergii* (4–6 cm) and *Pr. clarkii* (5–7 cm) were collected from the pond with high mortality in a farm in Jiangsu Province on 20 June 2018. In the same pond, some wild crustaceans, including *M. nipponense*, *M. superbum*, and some species of Cladocera, were also sampled for further analysis. Dead and dry bodies of *P. vannamei* (5–7 cm) were collected on the drained bottom of the adjacent pond, which suffered from a severe disease one month before in the farm.

### 2.2. DNA and RNA Extraction

Total DNA and RNA were extracted from 30 mg individual cephalothorax tissue of prawns, shrimp, or crayfish, or 30 mg multiple individuals of Cladocera by TIANamp Marine Animal DNA Kit and RNAprep pure Tissue Kit (TIANGEN Biotech, Beijing, China), respectively, according to the manufacturer’s instructions.

### 2.3. Pathogen Detection

DNA samples of *M. rosenbergii*, *P. vannamei*, *Pr. clarkii, M. nipponense*, *M. superbum*, and Cladocera were tested for White spot syndrome virus (WSSV), IHHNV, acute hepatopancreas necrosis disease-causing *Vibrio parahaemolyticus* (*Vp*_AHPND_), and DIV1 by real-time PCR methods. The RNA samples were tested for Yellow head virus (YHV), Infectious myonecrosis virus (IMNV), and CMNV by RT-real-time PCR methods. All the detection methods were recommended by the World Organization for Animal Health (OIE) [17] or developed before [5,18].

### 2.4. Histopathological Sections

Samples were fixed in Davidson’s alcohol-formalin-acetic acid fixative (DAFA) [19] for 24 h and then changed to 70% ethanol. Paraffin sections were prepared and stained with hematoxylin and eosin (H&E) staining according to the procedures of Bell and Lightner [19].

### 2.5. In Situ Digoxigenin-Labeled Loop-Mediated Isothermal Amplification (ISDL)

Samples were fixed in DAFA for 24 h and changed to 70% ethanol. The paraffin sections were then prepared and subjected to ISDL assays targeting the gene of the second largest subunit of DNA-directed RNA polymerase II of DIV1, according to the method adapted for DIV1 infection by Chen et al. [20].

### 2.6. Transmission Electron Microscopy (TEM)

Ultrathin sections of the white triangle tissue under cuticle at the base of rostrum (hematopoietic tissue) from diseased *M. rosenbergii* were prepared for observation with TEM. Small pieces of the hematopoietic tissue in <1 mm^3^ of sampled animals were fixed in TEM fixative (2% paraformaldehyde, 2.5% glutaraldehyde, 160 mM NaCl, and 4 mM CaCl_2_ in 200 mM PBS) (pH 7.2) for 24 h at 4 °C. Before ultrathin sectioning, the fixed tissues were secondarily fixed with 1% osmium tetroxide for 2 h, then embedded in Spurr’s resin and stained with uranyl acetate and lead citrate. Ultrathin sections were laid on collodion-coated grids and examined under a JEOL JEM-1200 electron microscope (Jeol Solutions for Innovation, Japan) operated at 80–100 kV in the Equipment Center of the Medical College of Qingdao University.

### 2.7. Quantitative Detection of DIV1 in Different Tissues of Naturally Infected M. rosenbergii

Total 15 moribund *M. rosenbergii* samples frozen at −80 °C were chosen to be defrosted and different tissues were separated, including hematopoietic tissue, antenna, uropods, pleopods, gills, pereiopods, muscle, and hepatopancreas. Total DNA was extracted from different tissues using TIANamp Marine Animal DNA Kit. The DIV1 loads in different tissues were detected by TaqMan probe-based quantitative real-time PCR (TaqMan qPCR) following our published method [5].

### 2.8. Relative Abundance of DIV1 in Different Tissues

In order to evaluate the distribution of DIV1 in different tissues, the relative abundance (*RA_i_*) of DIV1 in different tissues was calculated with the DIV1 load in each tissue (*L_i_*) to compare with the total load of DIV1 in the whole body, which were resulted from the sum of the DIV1 loads in all tested tissues (∑*L_i_*). The calculation is based on the following formula:

$$RA_{i}=\frac{L_{i}}{\sum L_{i}}$$

Significance analysis of relative abundance data between each of the two tissues was carried out using the *t*-test for heteroscedasticity hypothesis of two groups of samples with the add-in tool Data Analysis in Microsoft^®^ Excel^®^ 2016 MSO 64-bit.

## 3. Results

### 3.1. Observation of Clinical Signs of Diseaesd M. rosenbergii

According to on-farm inquiry, the investigated pond, about 1.5 ha in size was stocked with 45 postlarva/m^2^ of prawn *M. rosenbergii* in the middle of May and some juveniles of crayfish *Pr. clarkii* one week before. The adjacent pond stocked with shrimp *P. vannamei* suffered from an unknown disease and died out one month before. No disinfection or other effective control measure was taken for the ponds except drainage of the diseased shrimp pond. Symptoms of “white head” and “yellow gills” in the prawn population were observed two weeks before our arrival. During the first week, the disease developed slowly, but it became more and more severe in the second week. The moribund prawns lost their swimming ability and sank to the bottom of water and were rarely observed in shallow water. Moribund and dead prawns could be found every day in the diseased pond. In the following inquiry, we were told that the cumulative mortality was higher than 80%.

**Figure 1:**
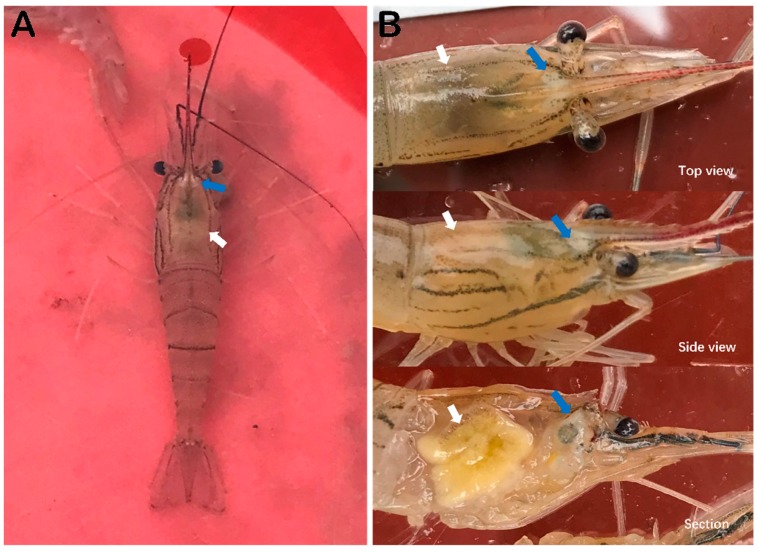
Clinical symptoms of *M. rosenbergii* (20180620) naturally infected with DIV1. (**A**) Overall appearance of a diseased prawn in water. (**B**) Close-up of cephalothoraxes. Blue arrows show white area under the cuticle at the base of rostrum. White arrows indicate hepatopancreas atrophy, color fading, and yellowing.

While the samples were taken and processed, it was observed that most of the caught *M. rosenbergii* prawns from the diseased pond exhibited obvious clinical signs, including a distinct white triangle area under the carapace at the base of rostrum, hepatopancreatic atrophy with color fading and yellowing in the section, empty stomach and guts (Figure 1A,B), and some moribund prawns were accompanied by slightly whitish muscle and mutilated antenna.

### 3.2. Pathogen Detection of Samples

A total of 20 DNA samples extracted from cephalothorax tissues of individual shrimp or multiple Cladocera were tested by real-time PCR or RT-real-time PCR. All samples were negative for WSSV, IHHNV, *Vp*_AHPND_, YHV, IMNV, and CMNV, but positive for DIV1. Samples of *M. rosenbergii* contained the highest DIV1 load range from 3.16 × 10^8^ to 9.83 × 10^8^ copies/μg-DNA. Samples of *M. superbum* contained the lowest DIV1 load (Table 1). 

### 3.3. Histopathology

**Figure 2:**
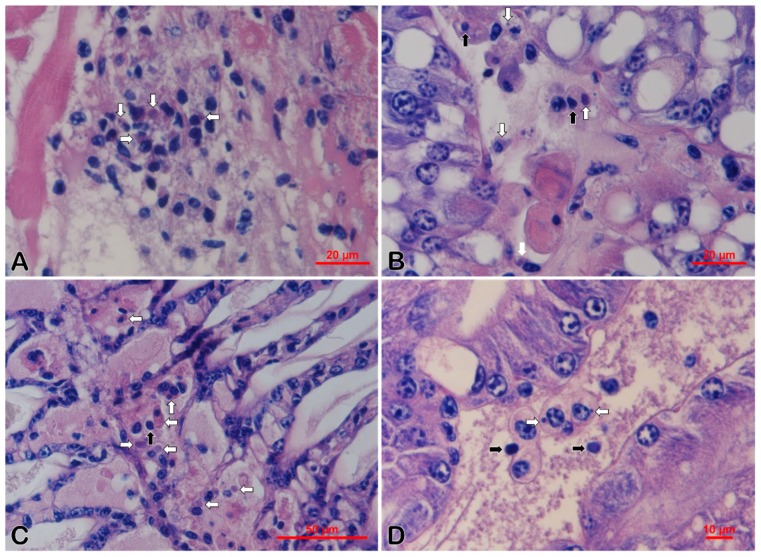
Histopathological features of Davidson’s alcohol-formalin-acetic acid fixative (DAFA) fixed *M. rosenbergii* (**A**–**C**) and *M. nipponense* (**D**) samples 20180620. White arrows show the eosinophilic inclusions and black arrows show the karyopyknotic nuclei. (**A**) Hematoxylin and eosin (H&E) staining of the hematopoietic tissue. (**B**,**D**) H&E staining of hepatopancreas. (**C**) H&E staining of gills. Bar, 20 μm (**A**,**B**), 50 μm (**C**), and 10 μm (**D**), respectively.

Histological examination of DAFA fixed samples showed that dark eosinophilic inclusions mixed with basophilic tiny staining and karyopyknosis existed in hematopoietic tissue (Figure 2A) and hemocytes in hepatopancreatic sinus (Figure 2B) and in gills (Figure 2C) of *M. rosenbergii*. For *M. nipponense*, dark eosinophilic inclusions and karyopyknosis were also observed in the hepatopancreas (Figure 2D). No typical histopathological feature was found in the tissue sections of *Pr. Clarkii* and Cladocera.

### 3.4. ISDL

**Figure 3:**
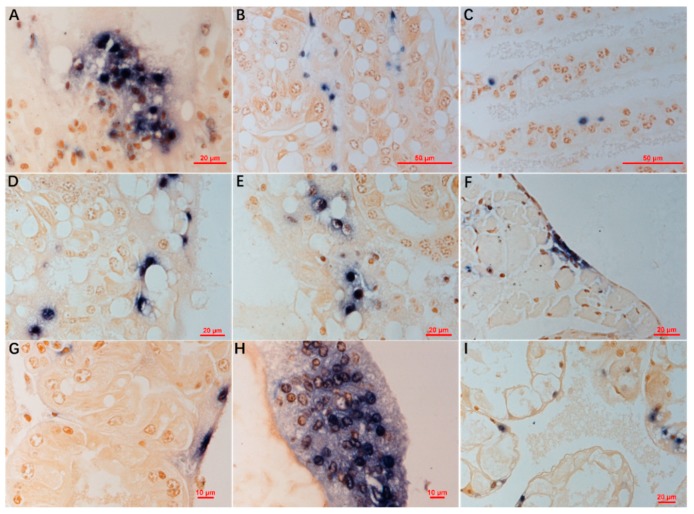
In situ digoxigenin-labeled loop-mediated isothermal amplification (ISDL) targeting the gene of the second largest subunit of DNA-directed RNA polymerase II of DIV1 on histological sections of *M. rosenbergii* (**A**–**F**), *M. nipponense* (**G**), and *Pr. clarkii* (**H**,**I**) samples 20180620. (**A**,**H**) Hematopoietic tissue; (**B**,**D**,**G**,**I**) hepatopancreas; (**C)** gills; (**E**) antennal gland; (**F**) ovaries. In (**A**–**C**,**H**), blue signals were observed in hematopoietic tissue, hemocytes in the sinus of the hepatopancreas, and in gills. In (**D**,**G**,**I**), blue signals exist in some hepatopancreatic R-cells and myoepithelial fibers. In (**E**), blue signals exist in the coelomosac epithelium. In (**F**), blue signals exist in the epithelium. Bar, 20 µm (**A**,**D**–**F**,**I**), 50 µm (**B**,**C**), and 10 µm (**G**,**H**), respectively.

ISDL results of *M. rosenbergii* showed that blue signals existed in hematopoietic tissue (Figure 3A), hemocytes in the hepatopancreatic sinus and in gills (Figure 3B,C), some R-cells, and myoepithelial fibers of the hepatopancreas (Figure 3D), coelomosac epithelium of antennal gland (Figure 3E), and epithelium of ovaries (Figure 3F). In addition, similar distribution of positive signals was also observed in the hepatopancreas of *M. nipponense* (Figure 3G) and hepatopancreas and hematopoietic tissue of *Pr. clarkii* (Figure 3H,I). No positive signals were observed in sections prepared from Cladocera, and uninfected prawn or crayfish.

### 3.5. TEM of Ultrathin Sections

**Figure 4:**
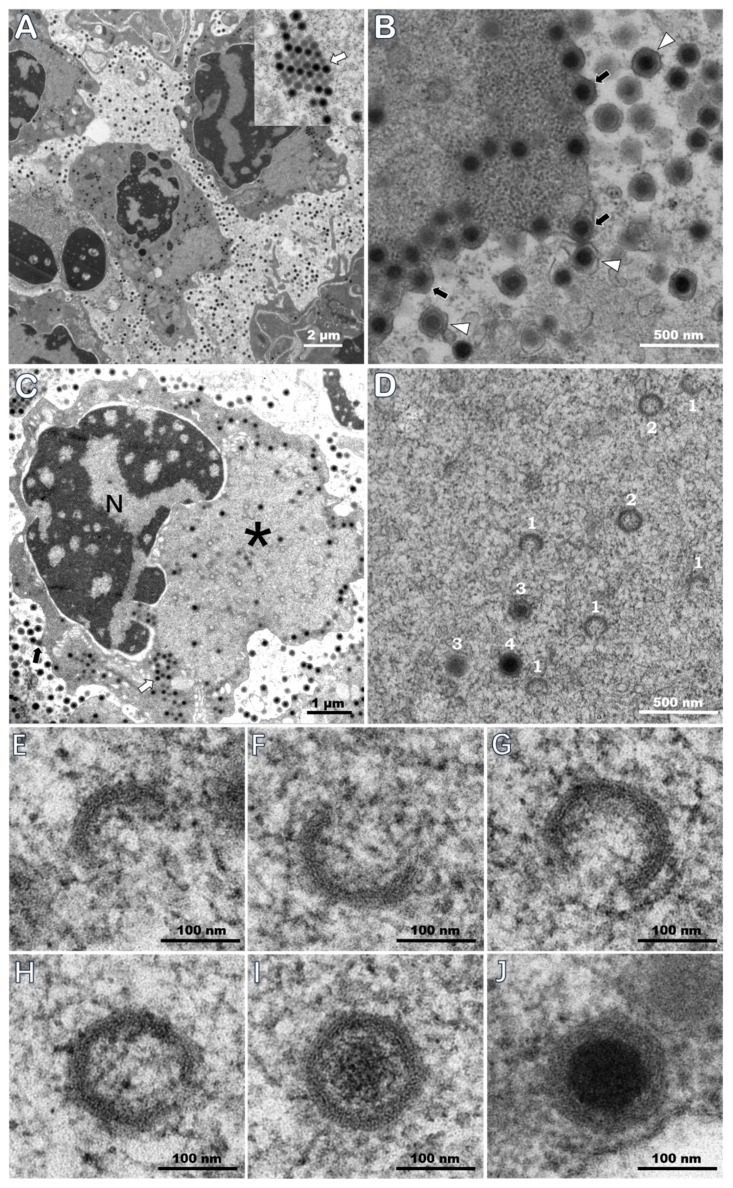
TEM of hematopoietic tissue of naturally infected *M. rosenbergii* samples 20180620. (**A**) A large numbers of virions in hematopoietic tissue. (**B**) DIV1 budded and acquired an envelope from the plasma membrane. (**C**) DIV1 replication and assembly in hematopoietic cells. (**D**) The stages of nucleocapsid assembly, which are indicated with numbers 1–3, and a complete nucleocapsid is indicated with number 4. The capsids at stage 2 and 3 should have a small opening at one vertex but may not be visible in the picture due to the ultrathin section. (**E**) Crescent-shaped structures. (**F**–**I**) As the assembling process continues, the crescent-shaped structure curves to form icosahedral capsids. (**J**) A mature virion with a dense core was eventually formed. N: nucleus; *: a large electron-lucent virogenic stroma; white arrows: paracrystalline array of viral particles; black arrows: budding virions; and white triangles: budded virions that acquired an envelope.

Visualization with TEM of ultrathin sections of the naturally infected *M. rosenbergii* revealed the presence of a large number of icosahedral particles with typical iridescent viral structure, both inside and outside hematopoietic cells in the tissue (Figure 4A). Non-enveloped virions were 166.3 ± 14.8 nm (*N* = 39) vertex to vertex (v-v), 149.4 ± 13.8 nm (*n* = 39) from face to face (f-f), and about 157.9 nm of an average equivalent diameter, with a nucleoid at 93.5 ± 9.9 nm (*n* = 39). At the margin of the cytoplasm, many virions were budding from the plasma membrane, and in budded virions, an outer viral envelope was acquired from the plasma membrane (Figure 4B). Virion formation took place in the cytoplasmic morphologically distinct regions, termed virogenic stromata, which were electron-lucent areas containing numerous immature and empty capsids, few mature virions, and were devoid of cellular organelles, with paracrystalline array of viral particles and budding virions in the same cell (Figure 4C). Assembly of nucleocapsid can be described in three progressive stages (Figure 4D), during which crescent-shaped structures of early capsid complexes subsequently assembled into spherical intermediates at stage 1 (Figure 4E–G, and indicated with 1 on Figure 4D), followed by formation of icosahedral capsids with a small opening at one vertex at stage 2 (Figure 4H, and indicated with 2 on Figure 4D) and recruitment of electron-dense nucleic acid at stage 3 (Figure 4I and indicated with 3 on Figure 4D). Complete nucleocapsids were observed in a fully filled state (Figure 4J, and indicated with 4 on Figure 4D).

### 3.6. Quantitative Detection of DIV1 in Different Tissues of Naturally Infected M. rosenbergii

A total of 120 tissues were separated from 15 naturally infected *M. rosenbergii* prawns. DIV1 loads in tissues of different prawns were examined with TaqMan qPCR. Copies of DIV1 per µg tissue DNA sample were converted to their logarithms for calculation of the geometric means and standard deviations of each tissue. The results showed that hematopoietic tissue samples contained an average DIV1 load of 10^(7.92 ± 0.91)^ copies/µg-DNA, which was the highest load of DIV1 in tissues tested. Antenna had a mean DIV1 load of 10^(7.84 ± 0.70)^ copies/µg-DNA, which approached hematopoietic tissue. Uropods, pleopods, gills, and pereiopods also had high loads of DIV1 above 10^7.6^ copies/µg-DNA of geometric means. Moreover, muscles and the hepatopancreas, as the vast majorities of cephalothorax and abdominal segments of the prawn, contained the lowest load of DIV1, which were at 10^(6.96 ± 0.57)^ and 10^(6.85 ± 0.72)^ copies/µg-DNA, respectively (Table 2). It was also noted that there were big differences of DIV1 loads by 3–4 orders of magnitude in all the tissues among different individuals, shown in the range column of Table 2.

### 3.7. Relative Abundance of DIV1 in Different Tissues

**Figure 5:**
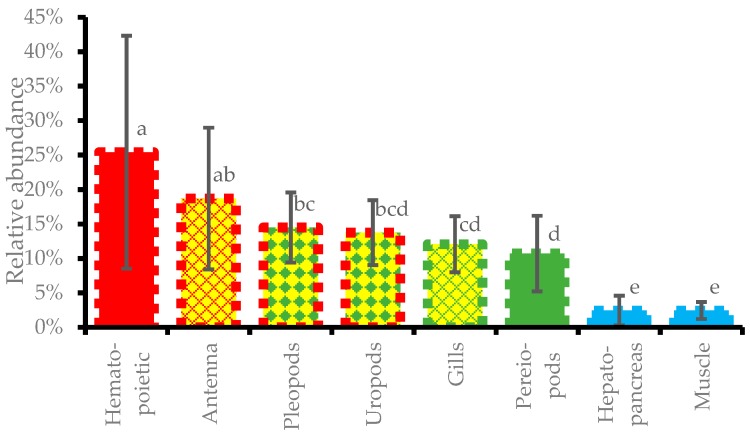
Relative abundance of DIV1 for different tissues of fifteen DIV1-infected *M. rosenbergii* samples. Columns without sharing of a same letter indicate significant difference of *p* < 0.05; columns without a same color indicate a highly significant difference of *p* < 0.01.

As the differences of DIV1 loads in tissues among different individuals reached 3–4 orders of magnitude, direct comparison on DIV1 loads in a specific tissue could be easily upset by a single sample with a very high DIV1 load. Therefore, relative abundance of DIV1 load based on a uniformization of the sum of DIV1 loads in all detected tissues was introduced to evaluate the distribution of DIV1 in different tissues. The relative abundance results showed that more than one-quarter of DIV1 relative load was in hematopoietic tissue, which contained the highest relative abundance at 25.4 ± 16.9%. Antenna, pleopods, and uropods contained significantly lower DIV1 relative abundances at 18.7 ± 10.3%, 14.5 ± 5.1%, and 13.8 ± 4.7%, respectively, more than hematopoietic tissue did (*p* < 0.05). Gills and pereiopods contained very significant lower DIV1 relative abundances at 12.1 ± 4.1% to 10.7 ± 5.5%, respectively, more than hematopoietic tissue did (*p* < 0.01). Hepatopancreas and muscle contained the very significantly lowest levels (*p* < 0.01) of the relative abundances of DIV1 at 2.44 ± 2.16% and 2.44 ± 1.24%, compared with all other tissues, respectively (Figure 5). When the total DIV1 load in all tissues fell between 10^8.24^ to 10^9.25^ copies/µg-DNA, most of the relative abundances of DIV1 in hematopoietic tissue fell into 12.2% to 35.9%, and even 71.7%; however, when the total DIV1 load in all tissues was lower than 10^7^ or higher than 10^9.5^ copies/µg-DNA, the relative abundances of DIV1 in hematopoietic tissue dropped to about 5% or lower, while the most DIV1 existed in antenna and pleopods.

## 4. Discussion

DIV1 belongs to a novel genus *Decapodiridovirus* accepted by ICTV, found independently in *C. quadricarinatus* and *P. vannamei* as CQIV and SHIV. Target surveillance was started in China in 2017 and revealed that the virus has been detected in 6 provinces out of the 13 surveyed provinces and caused massive economic losses [7].

As far as we know, there was no reported pathological information of *M. rosenbergii* infected with DIV1, but a PCR detection result showed that 5 of 33 cultured *M. rosenbergii* samples were DIV1 positive from 2014 to 2016 [2]. This study reported farmed *M. rosenbergii* and *Pr. clarkii*, cohabitating with some wild crustaceans, *M. nipponense*, *M. superbum*, and Cladocera in a pond in Jiangsu Province, suffered from severe mortality in June 2018, following the death of the *P. vannamei* population in the adjacent pond. Diseased giant freshwater prawns have exhibited typical symptom commonly, known as “white head” or “white spot”, since 2015 [15]. Real-time PCR results showed that all samples were negative for WSSV, IHHNV, *Vp*_AHPND_, YHV, IMNV, and CMNV, but positive for DIV1. Samples of *M. rosenbergii* contained the highest DIV1 loads, ranging from 3.16 × 10^8^ to 9.83 × 10^8^ copies/µg-DNA, which were higher than any other naturally infected species in this and earlier studies [5], indicating that the disease of *M. rosenbergii* in this case was caused by a severe infection with DIV1. This is the first confirmation of the causative agent of “white head” symptoms in farmed *M. rosenbergii*. In addition, in this case, DIV1 was also proved to be a natural pathogen to *P. clarkii* and *M. nipponense*. The disease firstly broke out and caused death of farmed *P. vannamei* population in the adjacent pond two weeks before clinical signs for prawns were found in the pond stocked with *M. rosenbergii*. This provided evidence that the transmitted disease cross ponds and species due to lack of biosecurity in the farm management.

Notably, susceptibilities of *M. rosenbergii*, *M. nipponense*, *P. vannamei*, and *Pr. clarkii* to infection with DIV1 and infection with WSSV are different. An earlier study revealed that *M. rosenbergii* and *M. nipponense* resisted infection with WSSV via intermuscular injection, but *Pr. clarkii* had high mortality at 94% [21]. Further challenge studies showed *M. rosenbergii* could be infected by injection with WSSV stains Thai-1 and Viet, however, the infectious dose to reach 50% endpoint in *M. rosenbergii* needed 20-fold and 400-fold more than that in *P. vannamei*, respectively [22]. The half lethal dose (LD50) of WSSV to *M. nipponense* by injection was 10^(3.84 ± 0.06)^ copies/g, which was about 1780-fold higher than that of *P. vannamei* at 10^(0.59 ± 0.22)^ copies/g [23]. It was demonstrated that *M. rosenbergii* could clear infectious WSSV after 5 to 50 days post-injection [24]. There is no report for the quantitative comparison on the virulence of DIV1 to different species yet. Based on the time course of mortality in intramuscular challenge with DIV1 (CQIV CN01), *P. vannamei* may be slightly more susceptible to DIV1 infection than *C. quadricarinatus* and *Pr. clarkii* [1]. The disease course observed in the farm for this study indicated that *M. rosenbergii* and *M. nipponense* have no tolerance to infection with DIV1. Many farms in Jiangsu, Guangdong, and Zhejiang Provinces [16,25], as well as in Southeast Asia [26,27] and Africa [28], have stocked ponds in polyculture mode with *M. rosenbergii* and *P. vannamei* or *P. monodon*. As *M. rosenbergii* has tolerance to infection with WSSV [21], the polyculture mode provides a profitable approach for farmers under the threat of WSSV. However, the emergence of DIV1 has shattered the vision and verified our earlier warning that polyculture with different species of crustaceans may bring risks for spread of disease, increase of susceptible species, and evolution of pathogens, based on our early surveillance on shrimp epidemiology [29].

*M. rosenbergii* and *P. vannamei* infected with DIV1 both exhibited hepatopancreatic atrophy with color fading on the surface and in the section, empty stomach, and gut. However, these symptoms are not distinctive, because empty stomach and gut also occurred in some other diseases, such as infection with WSSV [17,30], infection with Taura syndrome virus (TSV) and AHPND [17], and loss color of hepatopancreas is similar to the clinical feature of AHPND [17]. It is worth noting that “white head” is a typical clinical sign for on-site diagnosis of *M. rosenbergii* infected with DIV1. Xu et al. [1] reported that experimentally-challenged individuals of *Pr. clarkii* showed gross signs, such as cessation of feeding and flaccidity, at day-5 post-infection. In this case, cultured *Pr. clarkii* naturally infected with DIV1 contained a lower viral load (2.20 × 10^3^ to 1.57 × 10^4^ copies/µg-DNA) and suffered from an asymptomatic infection, because the juveniles of crayfish were stocked in the pond for only one week and some of them may have just been infected with the virus for a few days. Almost all of the *P. vannamei* shrimp in the adjacent pond died out in two weeks and the pond was abandoned by drainage. It was one month later when we arrived at the farm, and only several dried bodies of *P. vannamei* could be collected from the bottom of a drained pond, which could not be used for observation of gross signs.

Histopathological examination showed the existence of dark eosinophilic inclusions mixed or surrounded with basophilic staining and karyopyknosis in the hematopoietic tissues and hemocytes of gills and hepatopancreatic sinus in *M. rosenbergii*. Similar pathological features also existed in *M. nipponense* collected in this study and *Exopalaemon carinicauda* experimentally challenged with DIV1 (SHIV 20141215) [20]. The inclusions of DIV1 infection found in *P. vannamei* in previous research were described as basophilic, but the color of the inclusions on the published pictures was dark eosinophilic mixed with tiny basophilic staining, the same as in this study [2]. The eosinophilic inclusions presented in the cytoplasm of hemocytes or hematopoietic cells, which is very similar to some shrimp cases caused by the iridescent virus reported earlier [31,32], and the karyopyknosis is similar to some fish cases [33,34]. Compared with ISH in *P. vannamei* [2], ISDL also specifically indicated the existence and location of DIV1 in histologic sections. Blue signals were observed in hematopoietic tissue and hemocytes in the sinus of the hepatopancreas and gills in *M. rosenbergii*, *M. nipponense*, and *Pr. clarkii*, which is consistent with the ISH result in sections of infected *P. vannamei* [2]. However, differing from the results of ISH, positive signals resulted from ISDL were observed in some R-cells and myoepithelial fibers of the hepatopancreas, coelomosac epithelium of antennal glands, and the epithelium of ovaries, which indicated that besides hematopoietic tissues and hemocytes, DIV1 may also infect some other tissues at a lower level. More remarkably, blue signals of ISDL appeared in both the nucleus and cytoplasm of hematopoietic cells, hemocytes, and other infected cells. It signified that DIV1 may employ a replication strategy to include both nuclear and cytoplasmic stages, as Frog virus 3 (FV3), the typical species of genus *Ranavirus*, does [35,36,37]. It should be kept in mind that the amplification of target DNA in ISDL results in a similar climax quantity of amplified products, so that the density of positive signals does not relatively reflect the original quantity of the DNA target, and it can only indicate the presence and location of target viral nucleic acid in the cells of various tissues and organs [38].

TEM evidence also proved the diseased prawns *M. rosenbergii* were infected with DIV1 by viral morphology and cytopathology. The icosahedral morphology and intracytoplasmic location of virions are consistent with reports of DIV1 infections in *P. vannamei* [2] or in *C. quadricarinatus*, *Pr. clarkii*, and *P. vannamei* [4]. Non-enveloped virions showed values 166.3 ± 14.8 nm (v-v) and 149.4 ± 13.8 nm (f-f), with a nucleoid at 93.5 ± 9.9 nm, which were 7.7 nm, 5.8 nm, and 7.7 nm larger than previous study in *P. vannamei* [2]. The different size of virions may result from shrinkage of virions caused by the duration time of tissue samples stored in the TEM fixative and the dehydration and embedding procedures before ultrathin sectioning. Typical electron-lucent virogenic stroma was observed in hematopoietic cells of infected *M. rosenbergii*, containing immature, empty, and mature virions, with paracrystalline array of viral particles and budding virions in the same cell. The appearance and location of virogenic stroma were consistent with the dark eosinophilic inclusions observed on the H&E stained histopathological slides. Viral nucleic acid, intensive mature virions, and paracrystalline array embedded in and surrounding the virogenic stroma resulted in the dark eosinophilic and tiny basophilic staining. The progressive assembling stages, from the crescent shape complex to filled hexagonal nucleocapsids, are very similar to the forming process of Singapore grouper iridovirus (SGIV) nucleocapsid [39]. This observation was not reported for DIV1 in previous studies.

TaqMan qPCR specifically detected the highest loads of DIV1 in the lesion of whitish hematopoietic tissue at the base of rostrum of diseased *M. rosenbergii*. This data fills the gap in the previous study, which lacked the DIV1 load in hematopoietic tissue [5]. Unlike *M. rosenbergii*, *P. vannamei* has several very small hematopoietic tissues, which makes it hard to be seen and collected for quantitative detection of DIV1. Decapods have separate hematopoietic tissues located above the stomach and at the base of antennas, pereiopods, and gills as appendages of the cephalothorax [19]. That is why appendages under the cephalothorax also contained relatively higher DIV1 load than the main body. It is notable that the high loads of DIV1 were also detected in the appendages of the abdominal segments, including uropods and pleopods, which hinted that these appendages of abdominal segments may be attached with some hematopoietic cells. Muscle and hepatopancreas contained the lowest DIV1 loads, which may be due to the virus in hemocytes and hemolymph in these tissues. The relative abundances of DIV1 load in different tissues calculated based on the results of TaqMan qPCR could be used to quantitatively estimate the virus distribution for a tissue tropism study in comparison with different hosts. Interestingly, the naturally dried bodies of shrimp *P. vannamei* that had been dead for more than one month could still be detected as positive up to 7.19 × 10^6^ copies/µg-DNA. It indicated that dried shrimp bodies are still available for DIV1 detection.

## 5. Conclusions

This is a report of a natural occurrence of DIV1 in farmed giant freshwater prawn *M. rosenbergii*. All if the evidence resulting from symptom description, detection of known pathogens, histopathological and cytopathological observation, in situ DIG-labeled LAMP location, and quantitative detection of tissues consistently confirmed that the “white head” symptom of *M. rosenbergii* is the typical sign caused by infection with DIV1. Additionally, this study also provided evidence to add *Pr. clarkii* and *M. nipponense* as susceptible species to DIV1. The disease was likely transmitted from the adjacent pond stocked with *P. vannamei*, which had died out during the outbreak of infection with DIV1 due to lock of biosecurity in the farm management. The study provides a typical example of how DIV1 threaten the freshwater polyculture modes with different species of crustaceans, which we discourage.

## Figures and Tables

**Table 1:** Samples detected with the real time PCR for DIV1.

Species	Positive Samples	Total Samples	Geometric Mean (Copies/μg-DNA)	DIV1 Range (Copies/μg-DNA)
*M. rosenbergii*	5	5	10^(8.65 ± 0.21)^	3.16 × 10^8^–9.83 × 10^8^
*P. vannamei*	3	3	10^(5.96 ± 0.79)^	4.56 × 10^5^–7.19 × 10^6^
*M. nipponense*	3	3	10^(4.17 ± 1.68)^	1.30 × 10^3^–1.30 × 10^6^
*Pr. clarkii*	5	5	10^(3.82 ± 0.36)^	2.20 × 10^3^–1.57 × 10^4^
Cladocera	3	3	10^(1.10 ± 0.06)^	1.09 × 10^1^–1.43 × 10^1^
*M. superbum*	1	1	10^(1.04 ± 0.05)^	1.00 × 10^1^–1.18 × 10^1^

**Table 2:** DIV1 copies in different tissues detected in DIV1-positive prawns *Macrobrachium rosenbergii*.

Samples	n	Geometric Mean (Copies/μg-DNA)	Range (Copies/µg-DNA)
Hematopoietic tissue	15	10^(7.92 ± 0.91)^	8.11 × 10^4^–6.33 × 10^8^
Antenna	15	10^(7.84 ± 0.70)^	1.44 × 10^6^–1.03 × 10^9^
Uropods	15	10^(7.74 ± 0.71)^	6.45 × 10^5^–6.04 × 10^8^
Pleopods	15	10^(7.77 ± 0.69)^	4.77 × 10^5^–5.30 × 10^8^
Gills	15	10^(7.69 ± 0.66)^	4.45 × 10^5^–4.10 × 10^8^
Pereiopod	15	10^(7.62 ± 0.69)^	6.10 × 10^5^–1.05 × 10^9^
Muscle	15	10^(6.96 ± 0.57)^	1.87 × 10^5^–4.36 × 10^7^
Hepatopancreas	15	10^(6.85 ± 0.72)^	2.35 × 10^4^–3.18 × 10^7^
